# Transcriptome analysis reveals dynamic changes in coxsackievirus A16 infected HEK 293T cells

**DOI:** 10.1186/s12864-016-3253-6

**Published:** 2017-01-25

**Authors:** Jun Jin, Rujiao Li, Chunlai Jiang, Ruosi Zhang, Xiaomeng Ge, Fang Liang, Xin Sheng, Wenwen Dai, Meili Chen, Jiayan Wu, Jingfa Xiao, Weiheng Su

**Affiliations:** 1 National Engineering Laboratory for AIDS Vaccine, School of Life Sciences, Jilin University, Changchun, 130012 China; 2 BIG Data Center, Beijing Institute of Genomics, Chinese Academy of Sciences, Beijing, 100101 China; 3 Key Laboratory for Molecular Enzymology and Engineering, the Ministry of Education, School of Life Sciences, Jilin University, Changchun, 130012 China; 4 CAS Key Laboratory of Genome Sciences and Information, Beijing Institute of Genomics, Chinese Academy of Sciences, Beijing, 100101 China

**Keywords:** RNA-seq, miRNA-seq, Hand, foot and mouth disease, Coxsackievirus A16, SCARB2, Gene expression and regulation

## Abstract

**Background:**

Coxsackievirus A16 (CVA16) and enterovirus 71 (EV71) are two of the major causes of hand, foot and mouth disease (HFMD) world-wide. Although many studies have focused on infection and pathogenic mechanisms, the transcriptome profile of the host cell upon CVA16 infection is still largely unknown.

**Results:**

In this study, we compared the mRNA and miRNA expression profiles of human embryonic kidney 293T cells infected and non-infected with CVA16. We highlighted that the transcription of *SCARB2*, a cellular receptor for both CVA16 and EV71, was up-regulated by nearly 10-fold in infected cells compared to non-infected cells. The up-regulation of *SCARB2* transcription induced by CVA16 may increase the possibility of subsequent infection of CVA16/EV71, resulting in the co-infection with two viruses in a single cell. This explanation would partly account for the co-circulation and genetic recombination of a great number of EV71 and CVA16 viruses. Based on correlation analysis of miRNAs and genes, we speculated that the high expression of *SCARB2* is modulated by down-regulation of miRNA has-miR-3605-5p. At the same time, we found that differentially expressed miRNA target genes were mainly reflected in the extracellular membrane (ECM)-receptor interaction and circadian rhythm pathways, which may be related to clinical symptoms of patients infected with CVA16, such as aphthous ulcers, cough, myocarditis, somnolence and potentially meningoencephalitis. The miRNAs hsa-miR-149-3p and hsa-miR-5001-5p may result in up-regulation of genes in these morbigenous pathways related to CVA16 and further cause clinical symptoms.

**Conclusions:**

The present study elucidated the changes in 293T cells upon CVA16 infection at transcriptome level, containing highly up-regulated *SCARB2* and genes in ECM-receptor interaction and circadian rhythm pathways, and key miRNAs in gene expression regulation. These results provided novel insight into the pathogenesis of HFMD induced by CVA16 infection.

**Electronic supplementary material:**

The online version of this article (doi:10.1186/s12864-016-3253-6) contains supplementary material, which is available to authorized users.

## Background

Hand, foot and mouth disease (HFMD) threatens infants and children globally [1] and is caused by two main pathogens, CVA16 and EV71, both of which are members of the *Enterovirus* genus in the *Picornaviridae* family. Infection with EV71 commonly causes HFMD cases with severe symptoms, such as aseptic meningoencephalitis, brainstem encephalitis, myelitis, myocarditis and pulmonary edema [2]. By contrast, infection with CVA16 generally induces mild and self-limiting clinical symptoms, such as vesicular maculopapular rash, ulcers and pharyngitis [3], and only occasionally results in severe and fatal cases with central nervous system inflammation [4–8]. CVA16 infections have contributed to the majority of HFMD cases for decades, and co-infection and genetic recombination between CVA16 and EV71 have occurred frequently, likely leading to large disease breakouts and evolution of both viruses [9–13]. Therefore, greater attention should be paid to investigations of CVA16 infection mechanisms.

CVA16/EV71 infections begin with the binding of cellular receptors of the host cell, which were demonstrated to be human scavenger receptor class B member 2 (*SCARB2*) expressed extensively in tissues and human P-selectin glycoprotein ligand-1 (PSGL-1) expressed primarily in lymphocytes [14, 15]. The receptors mediate entry and subsequent replication of viruses. Our group described that SCARB2-overexpressing cell lines significantly increase their susceptibility to CVA16/EV71 of various genotypes [16], similar susceptibility increasing effect was also proved in PSGL-1 overexpressing cells [15, 17]. The infection of these viruses causes the disruption of cellular pathways and events. Host cell molecular alterations include relocalization of far upstream element binding protein 2 (FBP2), heterogeneous nuclear ribonucleoprotein A1 (hnRNP A1) and hnRNP K, phosphorylation of MAPK/ERK and PI3K/Akt pathways to assist virus replication [18–21], and cleavage of eukaryotic translation initiation factor 4G (eIF4G) by virus protease to inhibit the synthesis of host proteins and induce apoptosis [22]. Cell death and apoptosis in tissues subsequently result in HFMD symptoms.

In recent years, many studies on HFMD have been conducted at the transcriptome level, most of which use assay technologies to identify markers (genes or miRNAs) of EV71-related clinical symptoms in different tissues or cells [23–27]. However, the molecular mechanisms underlying vesicular rash formation caused by CVA16 infection and the potential central nervous system inflammation caused by enterovirus are still largely unknown. In this study, RNA-seq and miRNA-seq technologies were used to profile the transcriptome alterations in CVA16-infected and non-infected (CVA16-non-infected) cells, with the aim of gaining greater insight into the underlying mechanisms of CVA16-host interactions which may be highly relevant to disease pathogenesis in vivo.

## Results and discussion

### mRNA and miRNA expression profiles

We analyzed mRNA and miRNA expression profiles based on mRNA-seq and miRNA-seq for CVA16-infected as well as CVA16-non-infected cells to determine how CVA16 infection affects the function of a cell and the related regulatory mechanisms. In total, 88.1 M and 72.4 M mRNA-seq reads were generated for CVA16-infected and CVA16-non-infected cells, respectively. After trimming low quality bases, uniquely mapped rates of reads of these groups were both about 67% (Table 1). The saturation evaluation (Additional file 1) showed that the sequencing data were sufficient for analysis of gene expression. For CVA16-infected and CVA16-non-infected cells, 15.1 M and 7.5 M short reads of miRNA sequences were generated, respectively. We mapped miRNA clean short reads to a human pre-miRNA database and obtained 20.39 and 23.97% mapped rates and detected 447 and 446 miRNAs for CVA16-infected and CVA16-non-infected cells, respectively (Table 2).

**Table 1:** Mapping results of RNA-seq data for CVA16-infected and CVA16-non-infected cell samples

Sample ID	Number of reads			
	Raw data	After filtering	Mapped to genome	Uniquely mapped to genome
CVA16-infected	88,142,534	81,487,235 (92.45 %)	59,431,177 (72.93 %)	54,745,431 (67.18 %)
CVA16-non-infected	72,363,686	62,166,217 (85.91 %)	45,873,597 (73.79 %)	41,727,976 (67.12 %)

**Table 2:** Results of miRNA sequencing reads mapped to mirBase for CVA16-infected and CVA16-non-infected cell samples

	Number of reads		
	Raw data	After filtering	Mapped to miRBase
CVA16-infected	15,113,429	12,066,297 (79.84 %)	2,460,634 (20.39 %)
CVA16-non-infected	7,457,128	6,011,597 (80.62 %)	1,441,053 (23.97 %)

**Fig. 1:**
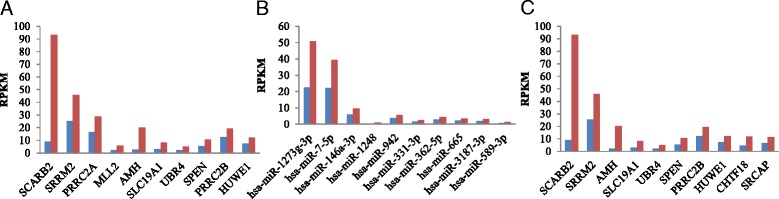
Differential gene expression between CVA16-non-infected and CVA16-infected cell samples. **a** Top 10 differentially expressed genes. **b** Top 10 differentially expressed miRNAs. **c** Top 10 differentially expressed target genes. *Blue* represents CVA6-non-infected. *Red* represents CVA6- infected

Among the identified 1954 differentially expressed genes, 1825 genes were up-regulated genes and 129 were down-regulated genes in CVA16-infected relative to CVA16-non-infected samples ((Differentially expressed genes are listed in Additional file 2, and the top 10 are shown in Fig. 1a). The 1954 differentially expressed genes were enriched in 822 third-level Gene Ontology (GO) terms (Additional file 3), mainly those involved in the regulation of cellular processes, cellular macromolecule metabolic processes and regulation of metabolic processes. The 51 differentially expressed miRNAs identified included 29 up-regulated miRNAs and 22 down-regulated miRNAs in CVA16-infected cells relative to CVA16-non-infected cells (Differentially expressed miRNAs are listed in Additional file 4, and the top 10 are shown in Fig. 1b). Therefore, 1323 differentially expressed target genes were predicted between CVA16-infected and CVA16-non-infected cells (Differentially expressed target genes are listed in Additional file 5, and the top 10 are shown in Fig. 1c).

### The expression regulation of *SCARB2* in CVA16-infected cells

**Fig. 2:**
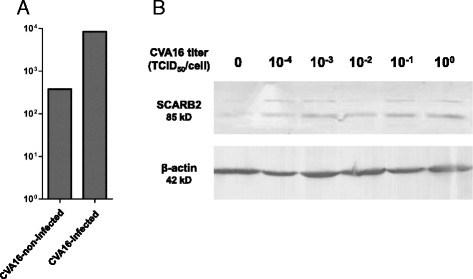
*SCARB2* mRNA and protein levels between CVA16-non-infected and CVA16-infected cell samples. **a** Fold change of *SCARB2*/*β-actin* mRNA copies detected by qRT-PCR. **b** *SCARB2* protein levels detected by Western blot

Virus infection is generally known to alter the expression of host genes and affect protein function. Thus, studying expression patterns of host genes may facilitate their use as diagnostic markers and therapeutic targets for diseases. CVA16 infection can cause clinical symptoms of HFMD, such as aphthous ulcers, cough and central nervous system dysfunctions. We found that the *SCARB2* gene was the most differentially expressed, as its level in CVA16-infected cells was 10 times higher than that of CVA16-non-infected cells (Fig. 1a). By quantitative RT-PCR (qRT-PCR) analysis, the copy number of *SCARB2* mRNA in CVA16-infected cells also was confirmed to be increased compared to that of the control (Fig. 2a). Furthermore, levels of the *SCARB2* protein in CVA16-infected cells were shown to be increased in a dose-dependent pattern (Fig. 2b). *SCARB2* is the cell membrane receptor for CVA16/EV71, and the up-regulation of its expression may increase the chance of co-infection with two viruses in a single cell. This finding would partly explain the large number of cases of EV71 and CVA16 co-circulation and genetic recombination. We also found that the miRNA hsa-miR-3605-5p, which targets the *SCARB2* gene, was down-regulated in CVA16-infected cells. The miRNA hsa-miR-3605-5p plays an important role in the processes of metal ion transport, monovalent inorganic cation transport, intracellular transport and transmembrane transport. Similarly, the major function of *SCARB2* in the cell is membrane transport [28]. Therefore, we inferred that the down-regulation of hsa-miR-3605-5p mediates the high expression of *SCARB2*.

### Function enrichment analysis of differentially expressed genes

**Fig. 3:**
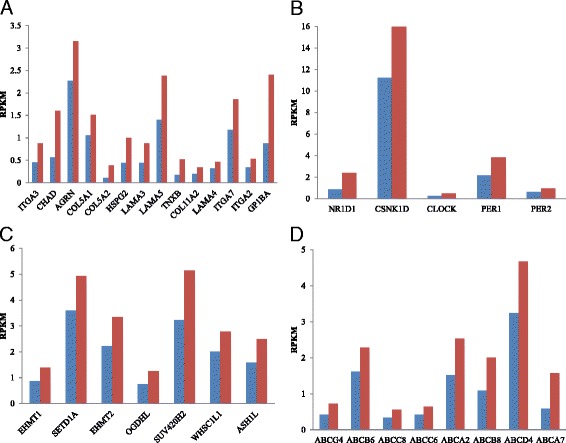
Differentially expressed genes between CVA16-non-infected and CVA16-infected cell samples clustered in four KEGG pathways. **a** ECM-receptor interaction pathway. **b** Circadian rhythm pathway. **c** ABC transporters pathway. **d** Lysine degradation pathway. *Blue* represents CVA6-non-infected. *Red* represents CVA6- infected

**Fig. 4:**
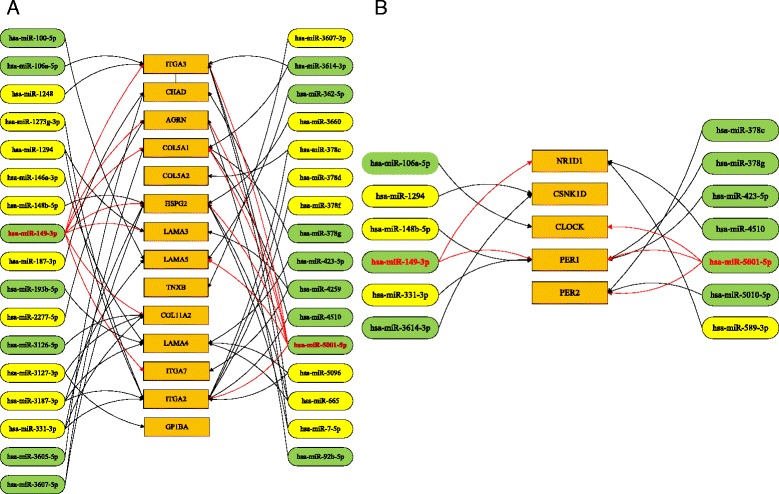
Regulation relation networks between differentially expressed target genes clustered in KEGG pathways and differentially expressed miRNAs. *Yellow* represents up-regulated miRNAs. *Green* represents down-regulated miRNAs. *Orange* represents differentially expressed genes clustered in KEGG pathways. **a** ECM-receptor interaction pathway. **b** Circadian rhythm pathway

These differentially expressed target genes between CVA16-infected and CVA16-non-infected cells were clustered into four functional pathways: ECM-receptor interaction, circadian rhythm, ABC transporters and lysine degradation (Table 3). We found that all of the differentially expressed target genes clustered in these four KEGG functional pathways were up-regulated (Fig. 3a-d). Some differentially expressed genes, regulated by miRNA, clustered in the ECM-receptor interaction pathway, which may be closely related to symptoms caused by CVA16 infection: aphthous ulcers, cough and myocarditis. Bakir-Gungor et al. [29] recently discovered that oral aphthous ulcers of Behcet’s disease may be correlated with the ECM-receptor interaction. Gunawardhana et al. [30] also found that inflammation of the airway is affected by the ECM-receptor interaction. The ECM in the heart and vascular wall consists of fibrous proteins and proteoglycans [31], which are important for maintenance of both the structure and function of the heart and vascular tissues [32]. Excessive deposition of ECM proteins has also been associated with many cardiac pathological entities such as myocardial fibrosis after myocardial infarction [33]. Therefore, we had reason to believe that the ECM-receptor interaction may be involved with symptoms of myocarditis, such as aphthous ulcers and cough, in some patients infected with CVA16. At the same time, we found that most of the differently expressed genes (9 out of 14) in the ECM-receptor interaction were modulated by the down-regulation of miRNAs hsa-miR-149-3p and hsa-miR-5001-5p (Fig. 4a). These genes regulated by hsa-miR-149-3p or hsa-miR-5001-5p, including *ITGA3*, *AGRN*, *COL5A1*, *HSPG2*, *LAMA3*, *LAMA5*, *COL11A2*, *ITGA7* and *ITGA2*, are all related to diseases.

**Table 3:** Clustering functional pathways of differentially expressed miRNA target genes

KEGG single pathway	Gene number	*P*-value
hsa04512:ECM-receptor interaction	14	0.00132
hsa04710:Circadian rhythm	5	0.005889
hsa02010:ABC transporters	8	0.014675
hsa00310:Lysine degradation	7	0.045685

Another observed enrichment pathway was the circadian rhythm pathway. Differentially expressed genes associated with the circadian rhythm pathway included *NR1D1*, *CSNK1D*, *CLOCK*, *PER1* and *PER2*. These genes were all up-regulated and related to the sleep phase syndrome and circadian clock. In other words, somnolence caused by CVA16 infection was correlated with the circadian rhythm pathway. Radomski et al. found that patients suffering from meningoencephalitis demonstrated circadian disruptions in cortisol, prolactin and sleep-wake rhythms. Sleeping sickness at the stage of meningoencephalitis manifests itself as a significant disturbance in the circadian rhythm of sleep-wakefulness [34]. Buguet et al. also found that the stage of meningoencephalitis in patients suffering from human African trypanosomiasis (HAT, sleeping sickness) represented a dysregulation of the sleep-wake cycle and sleep structure, rather than hypersomnia, and this dysregulation was accompanied by a circadian dysrhythmia of hormonal secretions. This finding shown that the meningoencephalitis is related to the circadian rhythm [35]. Other reports have shown that meningoencephalitis is one of the typical symptoms after EV71 infection [36–38]. Meningoencephalitis has also frequently appeared as one of the symptoms of Coxsackie B virus infection [38, 39]. As CV infection has been reported to cause lethargic encephalitis and mild paralysis in children [40, 41], we deduced that CVA16 interferes with intracellular circadian rhythms leading to somnolence with meningoencephalitis. Here, we found that 80% (4 out of 5) of the differentially expressed target genes clustered in the circadian rhythm pathway also are regulated by down-regulation of the miRNAs hsa-miR-149-3p and hsa-miR-5001-5p (Fig. 4b). Therefore, we deduced that the down-regulation of these two miRNAs may result in up-regulation of genes in morbigenous pathways related with CVA16 and further cause clinical symptoms.

## Conclusions

We examined the impact of infection with CVA16, a major causative pathogen of HFMD, using RNA-seq and miRNA-seq technologies to reveal the associated pathogenic mechanisms at the transcriptome level. We highlighted that the *SCARB2* gene was up-regulated by nearly 10-fold in infected cells compared to non-infected 293T cells at the transcriptional and translational levels. SCABR2 serving as a cellular receptor plays an important role in co-infections of EV71 and CVA16, and has-miR-3605-5p regulates the high expression of *SCARB2*. We found that differentially expressed miRNA target genes were mainly concentrated in the ECM-receptor interaction and circadian rhythm pathways, which may be related to clinical symptoms of patients infected with CVA16, such as aphthous ulcers, cough, myocarditis and somnolence of meningoencephalitis. Down-regulation of miRNAs hsa-miR-149-3p and hsa-miR-5001-5p may result in up-regulation of genes in these morbigenous pathways related to CVA16 infection, which further leads to clinical phenotypes.

## Methods

### Cells and viruses

293T cells (derived from human embryonic kidney cells) were cultured as monolayers in Dulbecco’s modified Eagle medium (DMEM) supplemented with 10% fetal calf serum (FCS). The CVA16 strain HN1129/CHN/2010 (B1b genotype, a gift kindly provided by the Henan Provincial Center for Disease Control and Prevention, China) was used for infection.

### CVA16 infections

Ninety percent confluent monolayers of 293T cells were infected with CVA16 at 1 TCID_50_ per cell. Two hours later, cells were washed twice with PBS and cultured in fresh DMEM (10% FCS). Fifteen hours later, the cells were harvested for transcriptome and qRT-PCR analyses.

### Next-generation sequencing of mRNA and miRNA and data processing

Sequence data were generated using Illuminna Hiseq 2000 following the manufacturer’s instructions for mRNA-seq and miRNA-seq. For mRNA-seq data, Fastx-toolkit (http://hannonlab.cshl.edu/fastx_toolkit/) was used to remove low quality reads and adapter sequences. Evaluation of mRNA-seq sequencing data showed that the sequencing quality was high (mean quality value was greater than 34). Reads (after removal of low quality reads and adapter sequences) were mapped to the human reference genome sequences (ENSEMBL62/GRCh37) with the Burrows-Wheeler Aligner (BWA) [42]. Only reads that were uniquely mapped to the reference genome sequences or were uniquely mapped to the junctions were chosen for subsequent analysis. Read densities for each gene were calculated by the number of uniquely mapped reads per kilobase per million mapped reads (RPKM). Differentially expressed genes were identified by DEGseq [43], which is an R-package available in Bioconductor (www.bioconductor.org/packages/2.7). A gene was considered to be significantly differentially expressed if the *p*-value and q-value were both less than 0.05.

For miRNA-seq data, if miRNA clean short reads (after removing low quality reads and adaptor sequences) were mapped to the whole human genome, both known miRNA and new miRNA could be obtained. However, the specificity of mapping short reads to the genome was very low due to the very short clean reads (<25 nt) and the complexity of the reference genome sequences. Therefore, in order to accurately observe known miRNA expression, we mapped clean short miRNA reads to human pre-miRNAs from the mirBase database (http://www.mirbase.org/) using SHRiMP2 software [44]. RPKM were computed to normalize the miRNA expression, and miRNAs were ignored when the number of mapped reads was less than 5. Differentially expressed genes were identified by DEGseq [43] using the same parameters as that for RNA-seq. To predict the gene target by miRNAs, two computational target prediction algorithms (RNAhybrid 2.1 [45] and miRanda 3.3 [46]) were used. The results predicted by both algorithms were combined, and the overlapping sequences were determined.

### Functional profiling of differentially expressed genes

The Database for Annotation, Visualization and Integrated Discovery (DAVID) (http://david.abcc.ncifcrf.gov/) was used to identify functional categories of differentially expressed genes. Following the instructions of the DAVID manual, differentially expressed genes in each sample were uploaded, and the function charts were generated. The functional groups with a *P*-value less than 0.05 and gene count greater than two were examined.

### qRT-PCR

Total RNA was extracted from cells using TRIzol reagent (Invitrogen). Target genes were amplified from 100 ng total RNA using a One Step SYBR Prime Script RT-PCR Kit (TaKaRa) and CFX96 real-time PCR detection instrument (Bio-Rad). *SCARB2* mRNA levels were analyzed with *β-actin* as the internal control. *SCARB2* primer: sense, 5′-GTACTGAGGCATTTGACTCCT-3′; antisense, 5′-AGTTCCCTGTAGGTGTATGGC-3′. *β-actin* primer: sense, 5′-CCACCATGTACCCAGGCATT-3′; antisense, 5′-CCGGACTCATCGTACTCCTG-3′.

### Western blot

293T cells were infected with CVA16 at 0, 10^−4^, 10^−3^, 10^−2^, 10^−1^ and 1 TCID_50_ per cell. After 2 h, the cells were washed twice with PBS and then cultured in fresh DMEM (10% FCS) and harvested after 15 h. The expression levels of *SCARB2* in 293T cells were detected by Western blot with β-actin as the internal control. After treatment with lysis buffer, proteins were subjected to 10% SDS-PAGE, and transferred to nitrocellulose membranes. After blocking with 5% nonfat milk and washing in Tris-buffered saline–Tween solution, membranes were incubated with goat anti-*SCARB2* primary antibody (1:2000, R&D Systems), followed by incubation with an alkaline phosphatase-conjugated rabbit anti-goat secondary antibody (1:5000, Sigma). The staining was carried out with NBT and BCIP solutions.

## Additional files

Additional file 1: RNA-Seq saturation curves. The horizontal axis represents number of reads. The left vertical axis represents the number of genes, and the right vertical axis represents the correlation coefficient. Saturation test results showed that the sequencing data were sufficient for analysis of differences in gene expression. (PPTX 49 kb)
Additional file 2: Differently expressed genes between CVA16-infected and CVA16-non-infected cell samples. (XLS 584 kb)
Additional file 3: GO functional enrichment of differentially expressed genes between CVA16-infected and CVA16-non-infected cell samples. (PPTX 68 kb)
Additional file 4: Differently expressed miRNA between CVA16-infected and CVA16-non-infected cell samples. (XLS 38 kb)
Additional file 5: Differently expressed target genes between CVA16-infected and CVA16-non-infected cell samples. (XLS 403 kb)

## Abbreviations

CVA16: Coxsackievirus A16
ECM: Extracellular membrane
EV71: Enterovirus 71
GO: Gene Ontology
HFMD: Hand, foot and mouth disease
RPKM: Reads per kilobase per million mapped reads
SCARB2: Scavenger receptor class B member 2
