# Supplementary figures and images for: Transcriptome analysis reveals dynamic changes in coxsackievirus A16 infected HEK 293T cells
Source: BMC Genomics. 2017 Jan 25;18(Suppl 1):933. doi: 10.1186/s12864-016-3253-6 (PMC5310284; doi:10.1186/s12864-016-3253-6)

## Slide 1
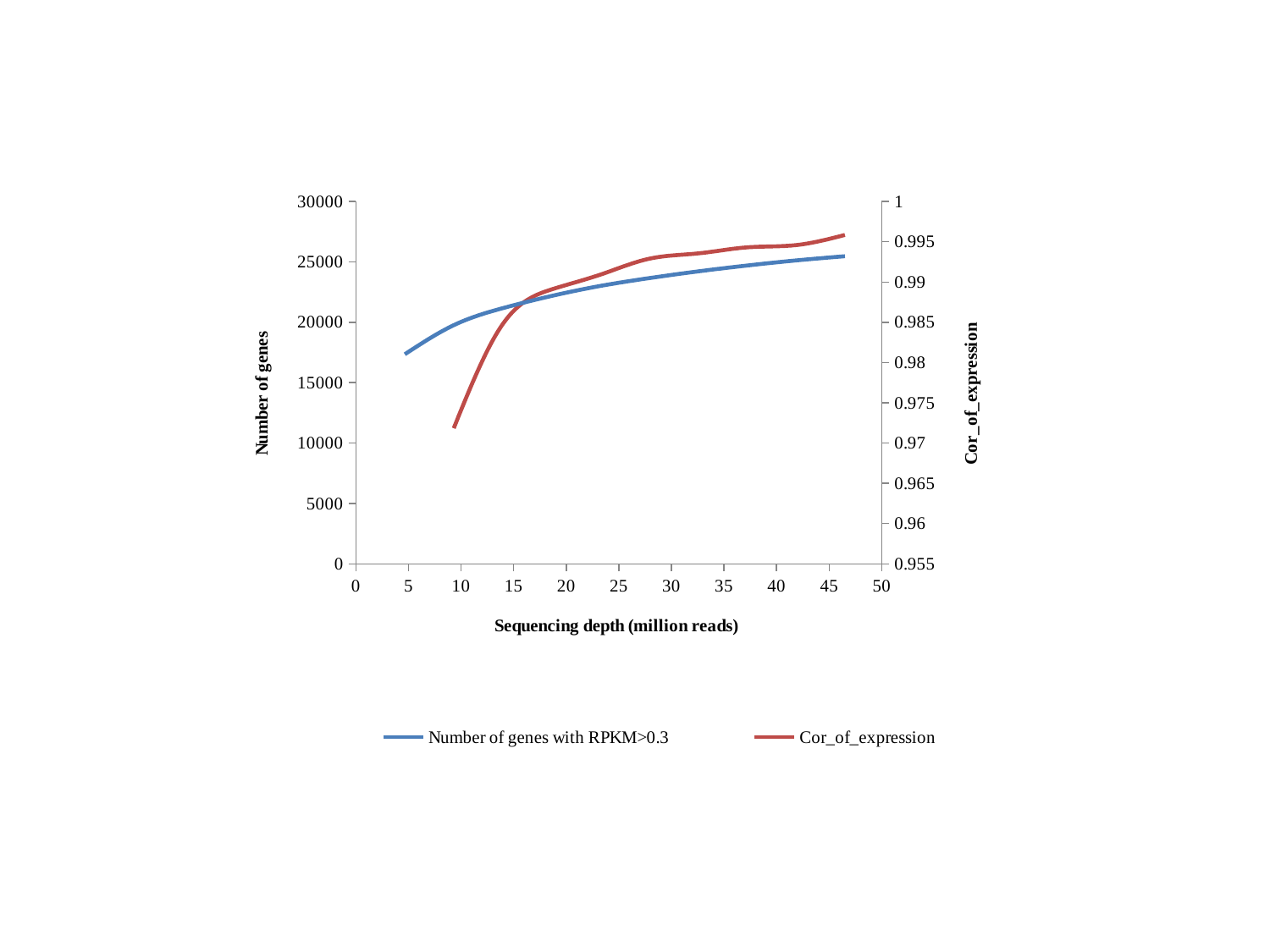

### Chart
| Category | Number of genes with RPKM>0.3 | Cor_of_expression |
|---|---|---|

Supplement: Additional file 1: — RNA-Seq saturation curves. The horizontal axis represents number of reads. The left vertical axis represents the number of genes, and the right vertical axis represents the correlation coefficient. Saturation test results showed that the sequencing data were sufficient for analysis of differences in gene expression. (PPTX 49 kb) [file 12864_2016_3253_MOESM1_ESM.pptx]
